# Supplementary figures and images for: Mitochondrial Small RNA Alterations Associated with Increased Lysosome Activity in an Alzheimer’s Disease Mouse Model Uncovered by PANDORA-seq
Source: Int J Mol Sci. 2025 Mar 26;26(7):3019. doi: 10.3390/ijms26073019 (PMC11988842; doi:10.3390/ijms26073019)

Heatmap of dyregulated genes in WT vs. AD mice

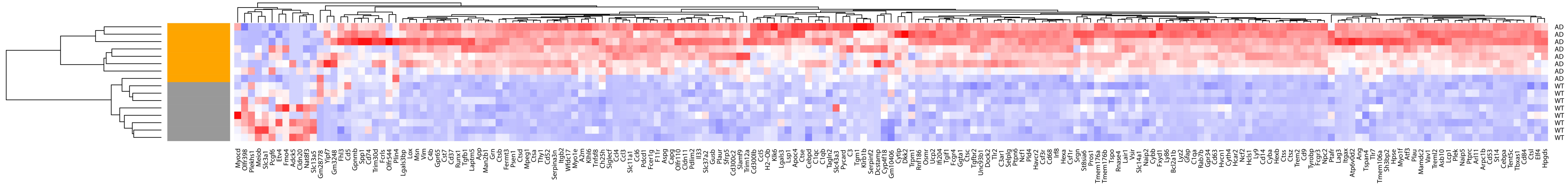

Supplement: Supplementary file 1 [file ijms-26-03019-s001.zip › ijms-3505925-supplementary.pdf]
